# Outstanding resistance and passivation behaviour of new Fe-Co metal-metal glassy alloys in alkaline media

**DOI:** 10.1371/journal.pone.0187567

**Published:** 2018-01-16

**Authors:** Khadijah M. Emran, Albandaree K. Al-Harbi

**Affiliations:** Chemistry Department, College of Science, Taibah University, Al-Madinah Al-Monawarah, Saudi Arabia; Ludwig-Maximilians-Universitat Munchen, GERMANY

## Abstract

The electrochemical behavior of the oxide layers on two metal-metal glassy alloys, Fe_78_Co_9_Cr_10_Mo_2_Al_1_ (VX9)and Fe_49_Co_49_V_2_ (VX50) (at.%), were studied using electrochemical techniques including electrochemical frequency modulation (EFM), electrochemical impedance spectroscopy (EIS) and cyclic polarization (CP) measurements. The morphology and composition of the alloy surfaces were investigated using X-ray photoelectron spectroscopy (XPS), scanning electron microscopy (SEM) and atomic force microscopy (AFM). The corrosion rate and surface roughness of both alloys increased as the concentration of NaOH in aqueous solution was raised. The presence of some protective elements in the composition of the alloys led to the formation of a spontaneous passive layer on the alloy surface. The higher resistance values of both alloys were associated with the magnitude of the dielectric properties of the passive films formed on their surfaces. Both alloys are classified as having outstanding resistance to corrosion, which results from the formation of a passive film that acts as an efficient barrier to corrosion in alkaline solution.

## 1. Introduction

The glassy alloys are considered to be particuarly interesting metallic materials because of exceptional properties, or combinations of physical and chemical properties, that distinguish metallic glassy alloys from conventional crystalline materials [1]. The formation of these metallic glasses by rapid melt quenching from the liquid state at a high cooling rate (10^6^ Ks^−1^) was first discovered in an Au-Si alloy system by Duwez et al. in 1960 [2]. However, Inoue found that for metallic alloys to have good glass-forming ability (GFA) the alloys should be composed of more than three elements. In addition, the component elements should have different atomic sizes and negative heats of mixing [3]. The first Fe-based bulk glassy alloys were prepared in 1995 [4] and, since then, a variety of Fe-based bulk glassy alloys have been prepared. Fe-based bulk metallic glasses (BMGs) are useful for industrial applications as magnetic, engineering, structural and surface coating materials due to the relatively low cost of the main alloying element (Fe). Iron confers outstanding properties, including high strength, a large elastic strain limit, and excellent wear and corrosion resistance, together with other remarkable engineering properties such as good ductility, high toughness and the inherent brittleness of the BMGs. Fe-based BMGs are amorphous structures with unique physical and chemical properties. These attractive qualities arise from a combination of certain properties that is not achievable with conventional crystalline alloys [5–8]. The most important thing that limits the use of these alloys is their susceptibility to corrosion. As is well known, alloying elements may inhibit corrosion. The presence of elements such as chromium and molybdenum in the alloy composition leads to greater corrosion resistance of Fe-based bulk metallic glasses [9]. Xu et al. studied the effect of addition of chromium in Cr-Fe metallic glasses and concluded that the Cr improved the mechanical properties and enhanced corrosion resistance. The stability of the chromium oxide increased with increasing chromium content. Addition of Cr enhanced resistivity to a certain extent, after which any further increase was ineffective (the corrosion resistance was stable above 29.4 at.% Cr) [10,11]. Gong et al. [12] investigated the corrosion behavior of Fe_65.5_Cr_4_Mo_4_Ga_4_P_12_C_5_B_5.5_ (at.%)bulk metallic glass in 3.0M NaOH solution. They found that the corrosion resistance of this alloy was better than its structural relaxation/crystallization counterparts. The (Fe_44.3_Cr_5_Co_5_Mo_12.8_Mn_11.2_C_15.8_B_5.9_)_98.5_Y_1.5_ bulk glassy alloy was studied in very basic solution (1.0M NaOH) by Gostin et al. [13]. This alloy exhibited a lower corrosion rate at higher pH.

In this study we explored the effect of NaOH concentration on passivation behaviour of two alloys (VX9 and VX50). Alloy resistivity can be explained according to the composition of the protective film formed in alkaline media, and the properties of these alloys make them very attractive for industrial application.

## 2. Experimental

Sheets of metal-metal glassy alloys Fe_78_Co_9_Cr_10_Mo_2_Al_1_ (VX9) and Fe_49_Co_49_V_2_ (VX50) (at.%) were supplied by Vacuumschmelze GmbH & Co. KG, Hanau, Germany. A surface area of 1 cm^2^ was used as the working area and each experiment was carried out with fresh sample. The test solution was an aerated solution of sodium hydroxide at different concentrations (0.1M, 0.25M and 0.5M) at ambient temperature (27°C). The electrochemical measurements were performed on an Interface 1000^™^ potentiostat/galvanostat (Gamry Instruments, Warminster, PA, USA). For accurate results, each experiment was repeated at least three times. The experimental data were analyzed using EFM140, EIS300 and DC105 software (Gamry Instruments). The electrochemical cell was comprised of three electrodes: the working electrode (sample), a saturated Ag/AgCl electrode and a platinum counter electrode.

The electrochemical frequency modulation (EFM) data were obtained using two frequencies, 2 and 5 Hz, with an amplitude of 10 mV, and the base frequency was 0.1 Hz at the open circuit potential (OCP). The electrochemical impedance spectroscopy (EIS) was conducted with an applied 10 mV sine wave and measurements were recorded over a frequency range of 800 kHz to 0.1 Hz. The cyclic polarization experiments were carried out after the impedance run in different concentrations of NaOH solution (0.1M, 0.25M and 0.5M) at a scan rate of 1.5 mVs^−1^. The potential forward sweep was from the cathodic to the anodic direction (−700 to 1200 mV) and the potential reverse sweep was from the anodic to cathodic direction over the same range.

Surface morphological characterization was performed after cutting the specimen at the passive region in the anodic polarization state. The alloy surface was examined by scanning electron microscopy (SEM, Superscan SS-550, Shimadzu, Japan) and atomic force microscopy (AFM, CP-II digital instrument, Veeco Instruments Inc., USA). Structural characterization was by X-ray photolectron spectroscopy (XPS) on an Axis Ultra DLD (Kratos Analytical Limited, Kyoto, Japan) with AlKα at 150 W of X-ray power in alkaline media.

## 3. Results and discussion

### 3.1 Electrochemical behaviour of Fe-Co metal-metal glassy alloys

**Fig 1:**
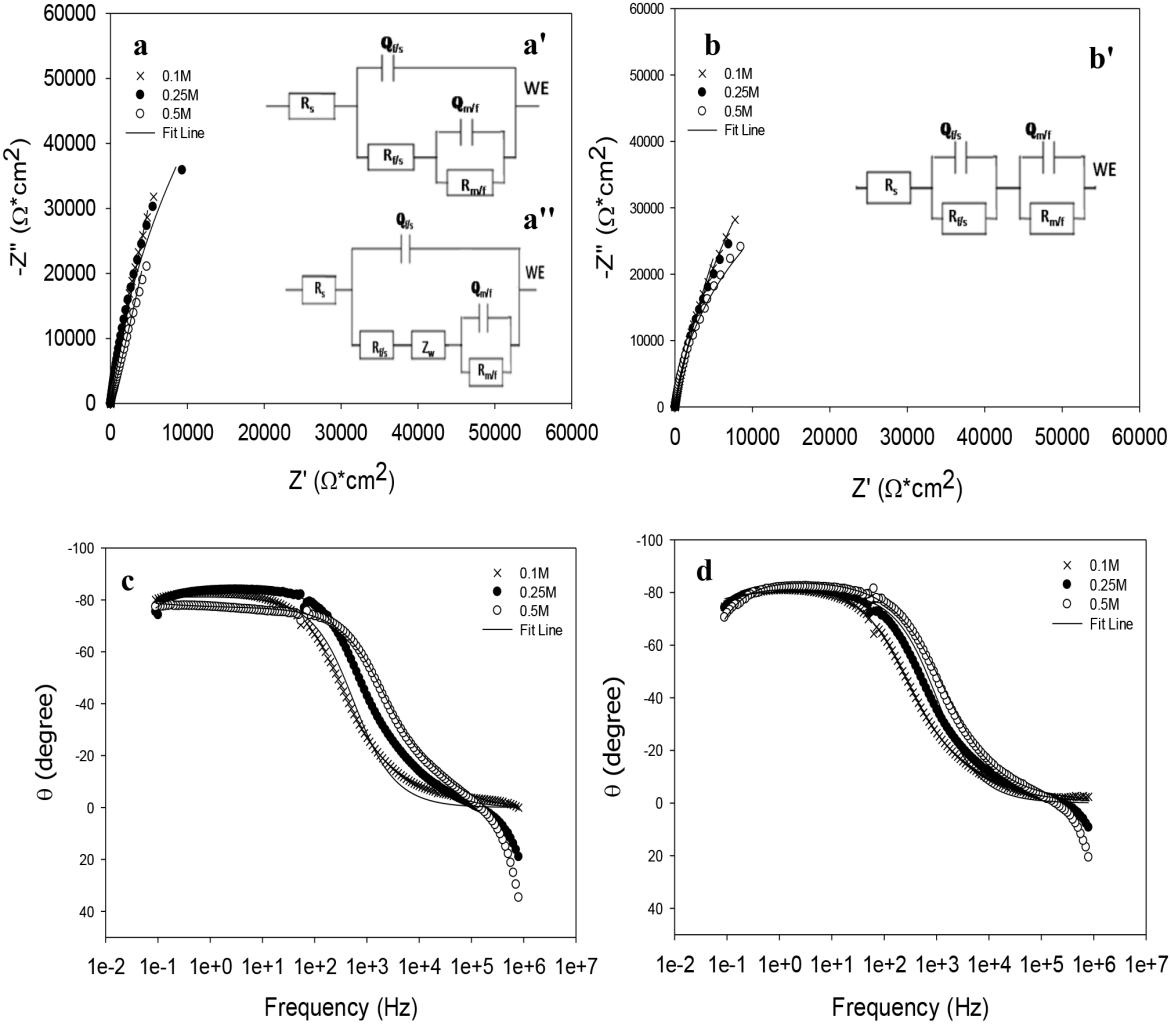
Nyquist plots and Phase angle plots for VX9 and VX50 alloys, respectively, at different concentration of NaOH solution, with the appropriate equivalent circuit. (a)The Nyquist plots for VX9 alloy at different concentration of NaOH solution. (a')The equivalent circuit for VX9 alloy at at 0.1 and 0.25M NaOH solution. (a'')The equivalent circuit for VX9 alloy at 0.5M NaOH solution. (b)The Nyquist plot for VX50 alloy at different concentration of NaOH solution. (b') The equivalent circuit for VX50 alloy at different concentration of NaOH solution. (c) The Phase angle plots for VX9 alloy at different concentration of NaOH solution. (d) The Phase angle plots for VX50 alloy at different concentration of NaOH solution.

Nyquist and Bode plots are shown in Fig 1(a–d) for VX9 and VX50 alloys at different concentrations (0.1M, 0.25M and 0.5M) of aqueous NaOH solution. As the NaOH concentration increased, the resistance of both alloys decreased (semicircle diameter decreased in Nyquist plots (Fig 1(a,b)) and the corrosion rate increased. The equivalent circuit (EC) illustrated in Fig 1(a') was used for VX9 alloy at 0.1M and 0.25M and that in Fig 1(a'') was used at 0.5M. For VX50 alloy, the EC in Fig 1(b') was used for fitting all Nyquist data. The fitting data are summarized in S1 Table (χ^2^ approximately 10^−4^–10^−6^).

The maximum phase angle, θ_max_, was around 85° (Fig 1(c,d)), suggesting that the electrochemical process occurring at high frequency favored the formation of passive film. The high resistance of VX9 alloy (S1 Table at the film/solution interface (R_f/s_) in NaOH solution at 0.1M and 0.25M (1.12 × 10^6^ and 282.6 × 10^3^ Ωcm^2^) results from formation of small pores in the passive film consisting of Cr_2_O_3_, MoO_2_ and Al_2_O_3_ (discussed in the XPS analysis). As these pores become larger, the passive film becomes more defective. The R_f/s_ decreased in 0.5M NaOH due to a high dissolution rate of Fe ions on the outer passive film. The passive film formed on the VX50 alloy surface consisted of two layers (Fig 1(b')). This suggests formation of a passive film in the form of a sandwich of cobalt oxide films consisting of an inner CoO layer and an outer Co_3_O_4_ layer. It can be concluded that the inner layer is able to grow to a certain thickness in the presence of the protective outer layer, probably because the outer layer (Co_3_O_4_) grows first. Consequently, the growth of the inner layer (CoO) is influenced by ionic diffusion through the outer layer, such that the thickness of the inner layer gradually increases after growth of the outer layer is completed [14,15]. The lowest resistance of VX9 alloy occurred at high NaOH concentration due to the presence of pores in the outer passive film (as shown in the SEM image). Fe ions are able to migrate faster through the inner film from the alloy at high NaOH concentration and Fe oxides are preferentially dissolved at the outer film, while Cr remains enriched in the passive film on the side of the alloy surface [16]. The CPE values (Q) in S1 Table indicate an increase of the corroded area as the NaOH concentration rose, causing thinning of the passive film or making it less protective. The alloy resistance fell 12.62-fold when the NaOH concentration was increased from 0.1M to 0.5M. From the results of EIS experiments, as shown in S1 Table, the resistance of the outer layer (R_f/s_) was much higher than the resistance of the inner layer (R_m/f_), suggesting that the outer layer was thicker than the inner layer. In general, the reason for the low resistance of the passive film formed on the VX50 alloy surface is the high level of iron compounds (FeOOH, Fe_2_O_3_ and Fe_3_O_4_). The iron oxides provide less protection of the alloy surface against corrosion because they behave as n-type semiconductors.

Finally, the resistance of VX9 alloy (1.12 × 10^6^ Ωcm^2^) was approximately five-fold (4.93) greater than the resistance of VX50 alloy (227.01 × 10^3^ Ωcm^2^) in 0.1M NaOH solution. This indicates that the passive film formed on the surface of VX9 alloy was more protective than that formed on the surface of VX50 alloy at low NaOH concentration. In addition, the resistance of the VX9 alloy (88.78 × 10^3^ Ωcm^2^) was 94.43% of that of the VX50 alloy (94.02 × 10^3^ Ωcm^2^) in the aggressive 0.5M concentration of NaOH S1 Table.

However, the high resistance values of the two alloys are associated with the magnitude of the dielectric properties of the passive films formed on their surfaces. As evidenced in S1 Table, the degree of inhomogeneity (n) for the two alloys at the film/solution interface was greater than 0.90, indicating capacitive behavior of the alloy surfaces.

**Fig 2:**
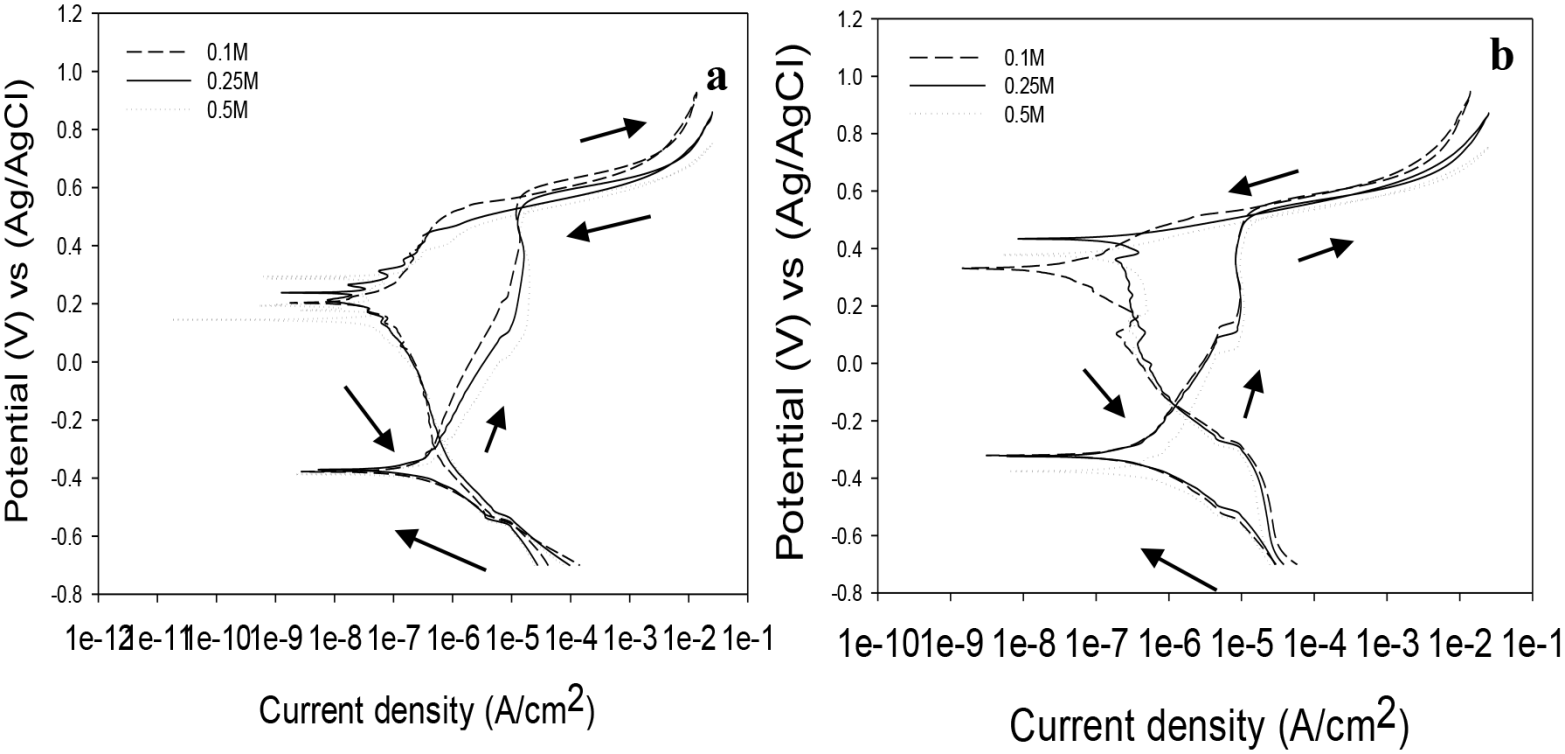
The cyclic polarization curves. (a)The cyclic polarization curves for VX9 at different concentration of NaOH solution. (b) The cyclic polarization curves for VX50 at different concentration of NaOH solution.

The cyclic polarization curves in Fig 2(a,b) for both VX9 and VX50 alloys show a wide range of spontaneous passivation with good protective properties in the NaOH solution due to the presence of protective elements in the composition of the alloys, such as Cr 10%, Mo 2% and Al 1% for the VX9 alloy and Co 49% for the VX50 alloy.

A very narrow positive hysteresis loop was observed for the VX9 alloy at all concentrations as shown in Fig 2(a), in contrast to the VX50 alloy. This was likely due to localized diffusion in the outer passive film, where the Fe migrates faster through the inner film at high NaOH concentration and preferentially dissolves at the outer film. The formation of a homogeneous passive film on the VX50 alloy surface resulted in the absence of these loops in NaOH solutions, as shown in Fig 2(b).

According to the data in S1 Table, the corrosion current densities (I_corr_), the corrosion rates (mmpy) and the I_pass_ values for both alloys increased as the concentration of the NaOH solution was increased. The porous passivating layer on the VX9 alloy surface in 0.5 M NaOH caused low stability for this layer, giving the highest value of I_pass_ (19.74 μAcm^−2^). Evidently, in 0.1M and 0.25M NaOH the passive film behaves as an efficient barrier against corrosion, suggesting healing of the pits. These results from the cyclic polarization measurements are in complete agreement with the EIS measurements.

Corrosion rates for the VX9 and VX50 alloys were < 0.02 mmpy at all concentrations, so they are classified as having outstanding resistance to the corrosion process [17]. All polarization curves for the VX9 and VX50 alloys were similar, which implied that each alloy had a similar corrosion mechanism in NaOH solution at different concentrations. It can also be inferred from the similar Tafel parameters (-b_c_) and the absence of a significant shift in the cathode branch (Fig 2(a,b)) that the cathodic reaction caused hydrogen evolution by the same mechanism.

The passive area decreased by about 89.58% for the VX9 alloy and 87.70% for the VX50 alloy as the NaOH concentration was increased from 0.1M to 0.5M. It can be observed from Fig 2(a,b) that another state was initiated in the forward sweep when the current density increased sharply, indicating that transpassive dissolution occurred. The E_b_ potential values were increased when the concentration of NaOH was decreased.

### 3.2 Electrochemical frequency modulation (EFM) measurements

**Fig 3:**
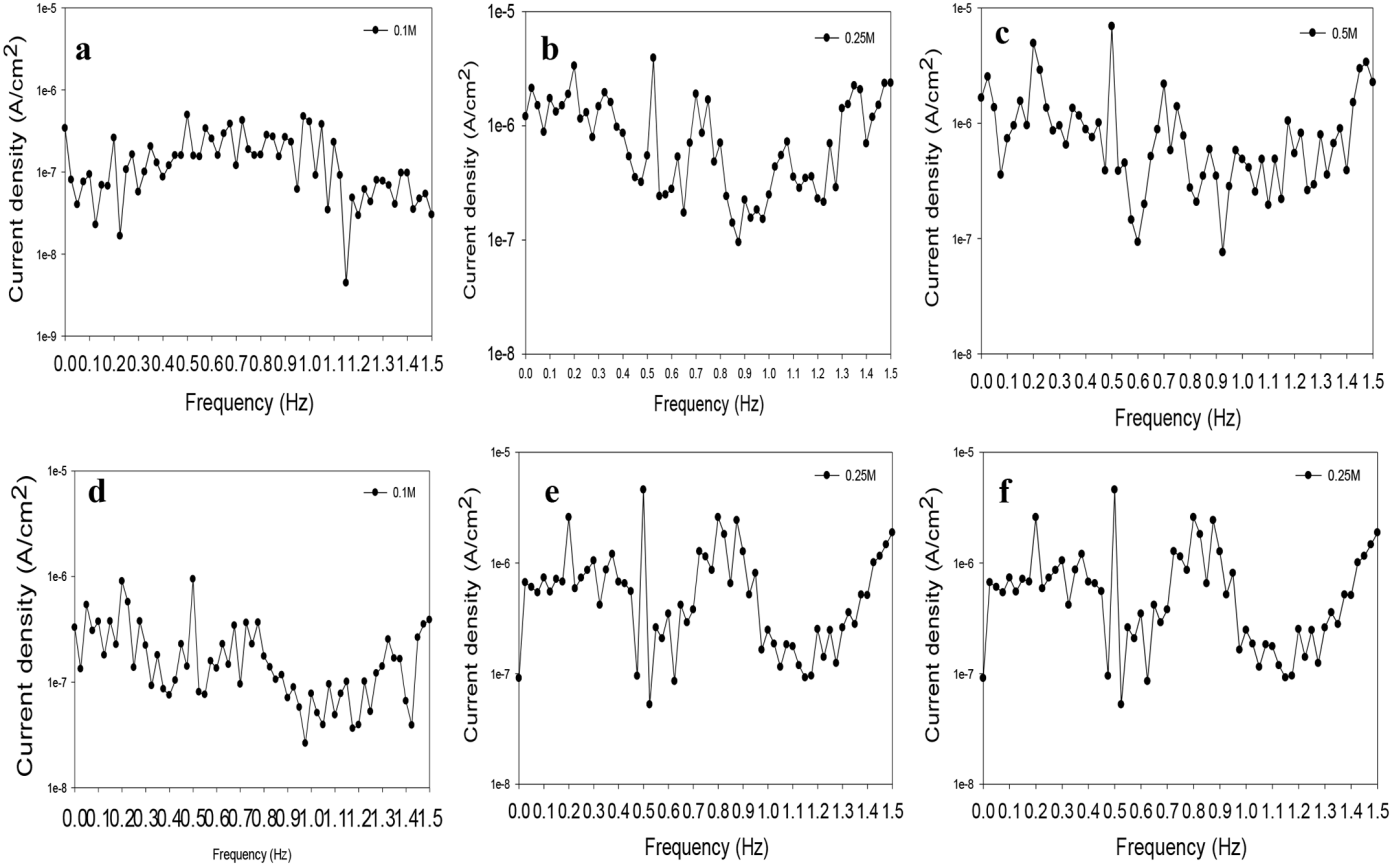
The intermodulation spectra. (a) The intermodulation spectrafor VX9 alloy at 0.1M NaOH solution. (b)The intermodulation spectrafor VX9 alloy at 0.25M NaOH solution. (c) The intermodulation spectrafor VX9 alloy at 0.5M NaOH solution. (d) The intermodulation spectrafor VX50 alloy at 0.1M NaOH solution. (e) The intermodulation spectrafor VX50 alloy at 0.25M NaOH solution. (f) The intermodulation spectrafor VX50 alloy at 0.5M NaOH solution.

The EFM method was used to monitor rates of corrosion and validate the corrosion rates measured by EIS and cyclic polarization. The EFM intermodulation spectra of VX9 and VX50 alloys are shown in Fig 3(a–f). S2 Table presents the electrochemical corrosion parameters obtained from EFM using the active model according to Eq 1.

$${\text{i}}_{\text{corr}}=\frac{{\text{i}}_{\omega_{1}\omega_{2}}{}^{2}}{2\sqrt[{}]{8{\text{i}}_{\omega_{1}\omega_{2}}{\text{i}}_{\text{2}\omega_{2}\pm \omega_{1}}-3{\text{i}}_{\omega_{2}\pm \omega_{1}}{}^{2}}}$$ (1)

The results indicate that the corrosion current density (I_corr_) and corrosion rate (mmpy) for both alloyswere increased as the NaOH concentration increased. The augmentation of the corrosion current density from 0.1M to 0.5M NaOH was about 3.36 times for VX9 alloy and 1.17 times for VX50 alloy. These data are in good agreement with those obtained from the DC and AC methods. The causality factors values, CF2 and CF3, suggest that the data obtained by this technique are reliable.

### 3.3 Passive film composition

The XPS spectraof Co 2p_3/2_ for the VX9 alloy and V 2p_3/2_ for the VX50 alloy before and after exposure to NaOH solution indicated that the concentrations of cobalt and vanadium in the film surfaces were negligibly low.

**Fig 4:**
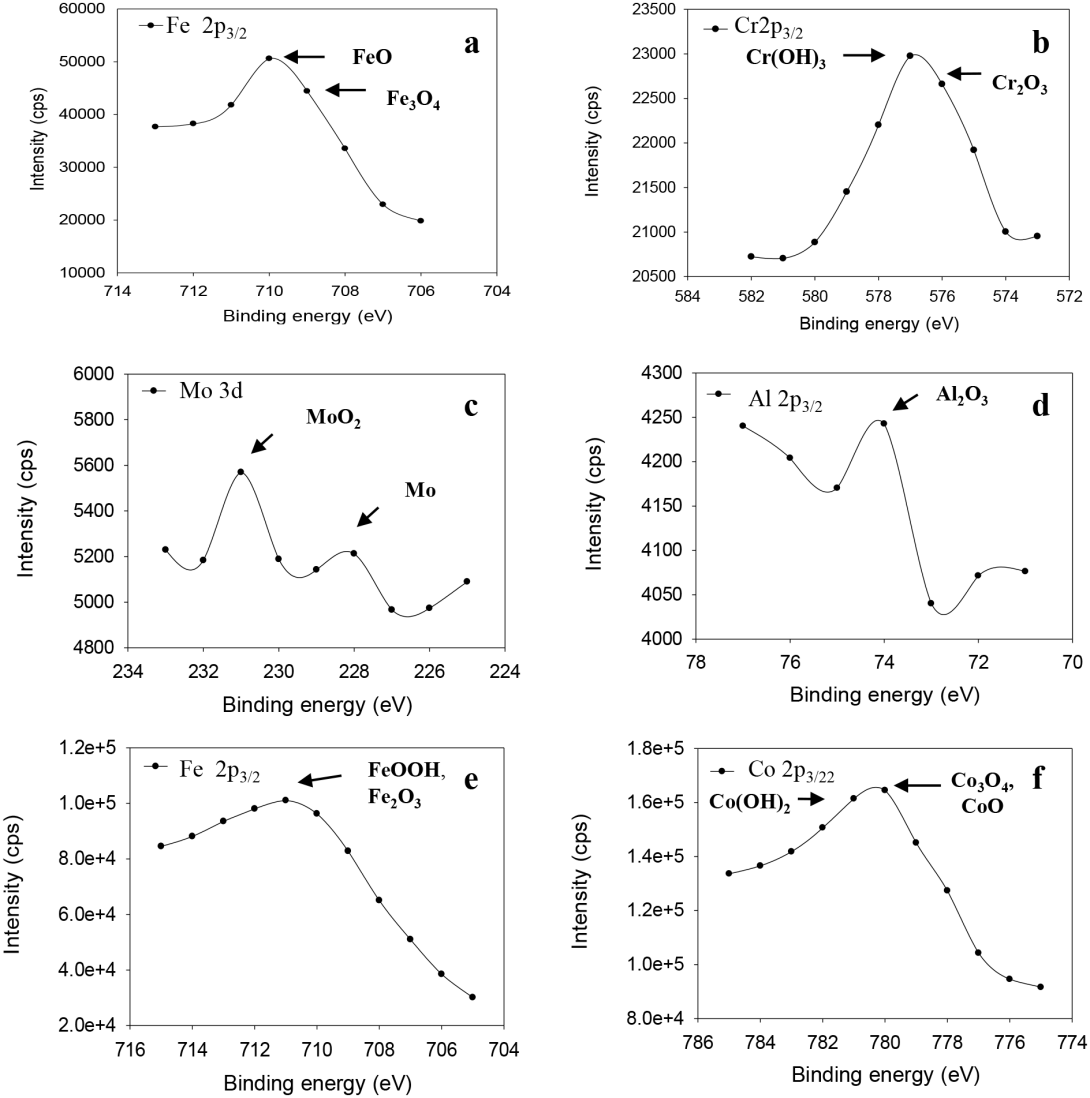
The XPS deconvolution spectra. (a) The XPS deconvolution of Fe 2p3/2 spectra for VX9 alloy in NaOH solution. (b) TThe XPS deconvolution of Cr2p3/2 spectra for VX9 alloy in NaOH solution. (c) The XPS deconvolution of Mo 3d spectra for VX9 alloy in NaOH solution. (d) The XPS deconvolution of Al 2p3/2 spectra for VX9 alloy in NaOH solution. (e) The XPS deconvolution of Fe 2p3/2 spectra for VX50 alloy in NaOH solution. (f) TThe XPS deconvolution of Cr2p3/2 spectra for VX50 alloy in NaOH solution.

In the iron spectrum, as shown in Fig 4(a), a broad binding energy peak corresponding to Fe^ox^2p_3/2_ was observed for VX9 alloy after exposure to the NaOH solution. The highest binding energy peaks, located at 710 and 709 eV, indicated the presence of Fe^2+^ in the form of iron oxides (FeO and Fe_3_O_4_, respectively) on the surface [18,19]. As illustrated in Fig 4(b), other peaks located at 576.3 and 577.1 eV for Cr^ox^2p_3/2_ corresponded to Cr_2_O_3_ and Cr(OH)_3_, respectively, on the surface of the VX9 alloy. These chromium compounds indicate high resistance of the passive film [18,19]. The Mo 3d spectrum (Fig 4(c)) contained two peaks corresponding to Mo^m^ 3d_5/2_ and Mo^ox^ 3d_3/2_ situated at 228 and 231 eV, indicating the presence of the metallic species, Mo and MoO_2_, respectively [20]. The highest binding energy peak in the Al^ox^ 2p spectrum (Fig 4(d)) located at 74 eV indicates the presence of Al_2_O_3_ on the surface of the VX9 alloy [18]. These oxides signify high corrosion resistance of the passive film.

The peak corresponding to Fe^ox^2p_3/2_ for VX50 alloy located at 711 eV (Fig 4(e)) relates to the presence of Fe^3+^ as iron oxide and oxyhydroxide species (FeOOH, Fe_3_O_4_ and Fe_2_O_3_) in the passive layer [18,19]. The highest binding energy peaks in the Co 2p_3/2_ spectrum (Fig 4(f)), located at 779.9, 780 and 781 eV, correspond to CoO, Co_3_O_4_ and Co(OH)_2_, respectively, on the VX50 alloy surface, of which Co_3_O_4_ is considered to be very effective in passive films [18,19]. The peak in the O^ox^ 1s spectrum at 530 eV illustrated the presence of O^2−^ ion or an M–O bond, and the peak at 531 eV is due to OH^−^. These peaks can be attributed to the formation of oxide or hydroxide components, respectively.

### 3.4 Surface characterization of the alloy in different concentrations of NaOH

**Fig 5:**
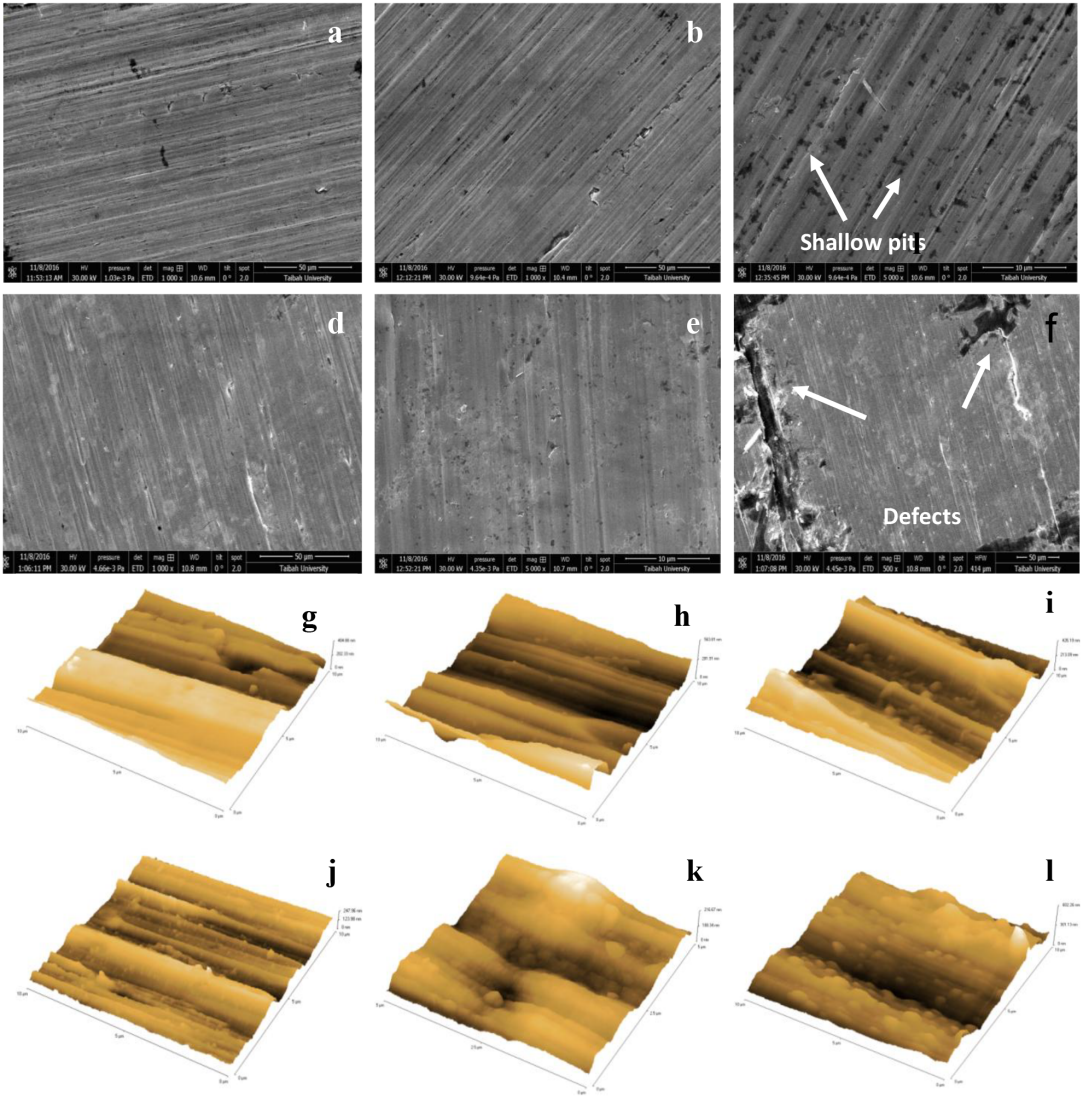
The SEM micrograph at critical immersion time and the AFM images. (a) The SEM micrograph for VX9 alloy at 0.1M NaOH solution. (b) The SEM micrograph for VX9 alloy at 0.25M NaOH solution. (c) The SEM micrograph for VX9 alloy at 0.5M NaOH solution. (d) The SEM micrograph for VX50 alloy at 0.1M NaOH solution. (e) The SEM micrograph for VX50 alloy at 0.25M NaOH solution. (f) The SEM micrograph for VX50 alloy at 0.5M NaOH solution. (g)The AFM images for VX9 alloy at 0.1M NaOH solution. (h)The AFM images for VX9 alloy at 0.25M NaOH solution. (i) The AFM images for VX9 alloy at 0.5M NaOH solution. (j) The AFM images for VX50 alloy at 0.1M NaOH solution. (k)The AFM images for VX50 alloy at 0.25M NaOH solution. (l) The AFM images for VX50 alloy at 0.5M NaOH solution.

Comparison of the surfaces of the two alloys (VX9 and VX50) before and after exposure to NaOH solution (0.1M–0.5M) is shown in Fig 5(a–f). The surfaces of the as-received alloys were uniform before exposure to NaOH solution. It is clear that a passive layer was formed on the surface of VX9 alloy, protecting the surface, at low concentrations of NaOH (0.1M and 0.25M) as illustrated in Fig 5(a,b, respectively). Subsequently, the thickness of this passive layer was decreased when exposed to the higher concentration 0.5M NaOH solution, accompanied by a decrease of the resistance. In addition to thinning, the surface consisted of shallow pits reflected by the sharp increase of capacitance (Q_s/f_) at 0.5M (Fig 5(c)).

The thickness of the passive layer on the VX50 alloy surface also decreased as the concentration of the NaOH solution increased. This thinning was accompanied by the appearance of defects in 0.5M NaOH, as shown in Fig 5(f).

The surfaces of the VX9 and VX50 alloys were examined by AFM after exposure to NaOH solution to determine the surface roughness at different concentrations (0.1M, 0.25M and 0.5M) as shown in Fig 5(g–l).

The AFM parameters are summarized in S3 Table. In general, the average roughness (R_a_) of VX9 and VX50 alloys surfaces was directly proportional to the NaOH concentration and increased about 2.32-fold for VX9 alloy and 1.72-fold for VX50 alloy between 0.1M and 0.5M NaOH. The data in S3 Table can be summarized as follows: at low concentration (0.1M and 0.25M), (a) the total roughness (R_*t*_) of VX9 alloy was less than that of VX50 alloy, (b) the valley depth (R_*v*_) on the surface of the VX50 alloy was greater than that on the VX9 alloy; at high concentration (0.5M) (c) it was evident that the highest peak (R_*p*_) was recorded on the VX9 alloy surface, (d) the deepest valley was observed on the surface of the VX9 alloy (168.2 nm, compared with 156.7 nm on the surface of the VX50 alloy). This indicates that the VX9 alloy surface was significantly affected at the high concentration of NaOH. These results are in agreement with the electrochemical measurements.

## Conclusions

The passivation behavior of Fe_78_Co_9_Cr_10_Mo_2_Al_1_ and Fe_49_Co_49_V_2_ glassy alloys was investigated in NaOH solution (0.1M, 0.25M and 0.5M). As the concentration of NaOH was increased, theresistance of both alloys decreased and the corrosion rate increased. Consequently, the roughness of the VX9 and VX50 alloy surfaces was directly proportional to the concentration of NaOH in solution, in line with the decreased resistance. The passive film formed on the VX50 alloy surface was a sandwich-type of cobalt oxide films consisting of an inner CoO layer and an outer Co_3_O_4_ layer. The resistivity of the two alloys was associated with the magnitude of the dielectric properties of the passive films formed on their surface.

## Supporting information

S1 Table Results from impedance and cyclic polarization measurements for the both alloys in different concentrations of NaOH solution. (TIF)

S2 Table Electrochemical corrosion parameters of the VX9 and VX50 alloys using EFM in technique at different concentrations of NaOH solution using the active model. (TIF)

S3 Table The AFM parameters for VX9 and VX50 alloy surfaces in different concentrations of NaOH solution. (TIF)
