# Supplementary figures and images for: Outstanding resistance and passivation behaviour of new Fe-Co metal-metal glassy alloys in alkaline media
Source: PLoS One. 2018 Jan 16;13(1):e0187567. doi: 10.1371/journal.pone.0187567 (PMC5770034; doi:10.1371/journal.pone.0187567)

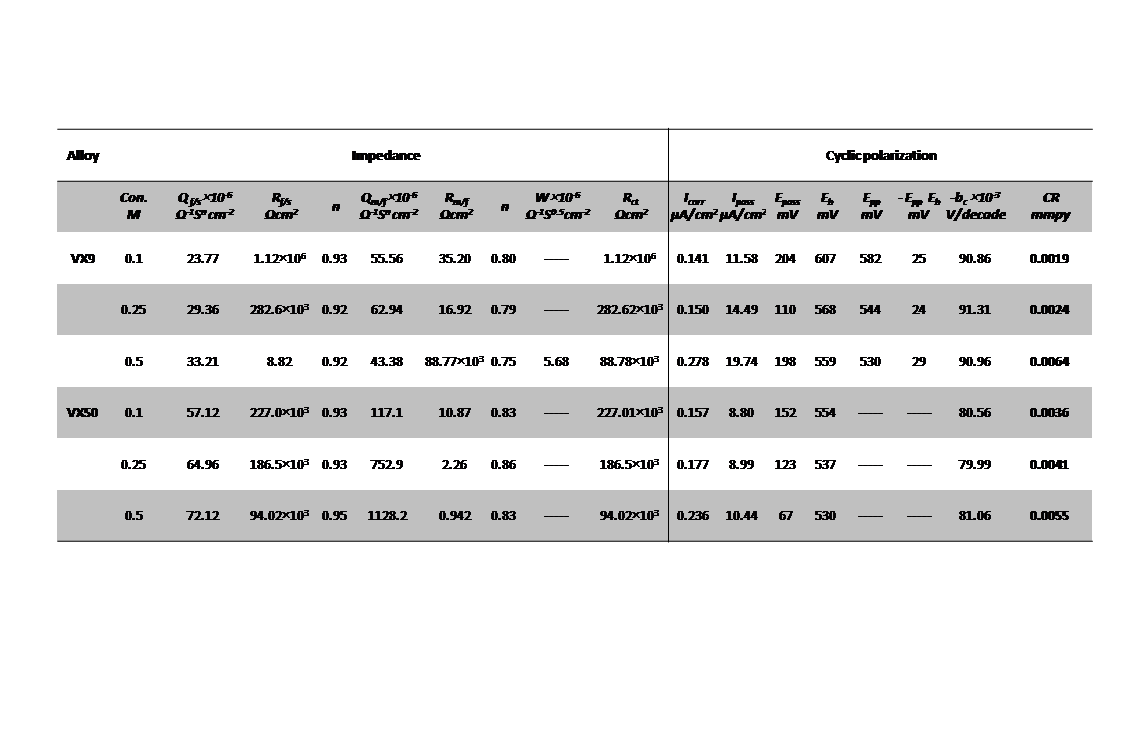

Supplement: S1 Table — (TIF) [file pone.0187567.s001.tif]

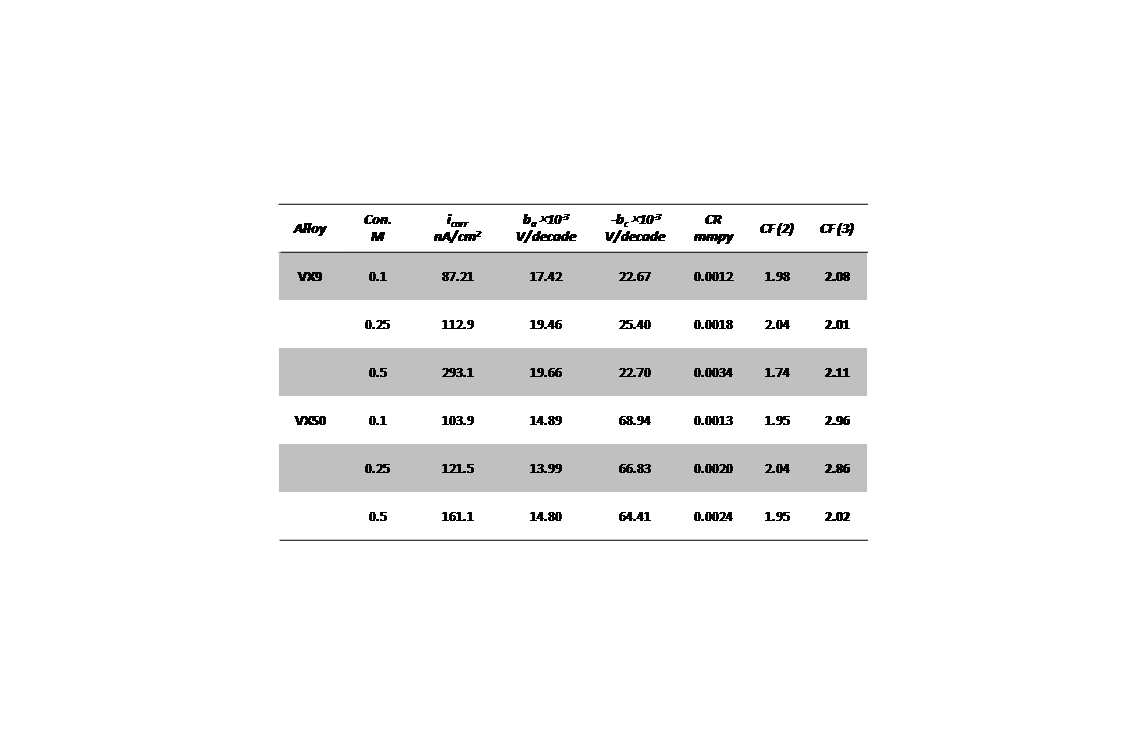

Supplement: S2 Table — (TIF) [file pone.0187567.s002.tif]

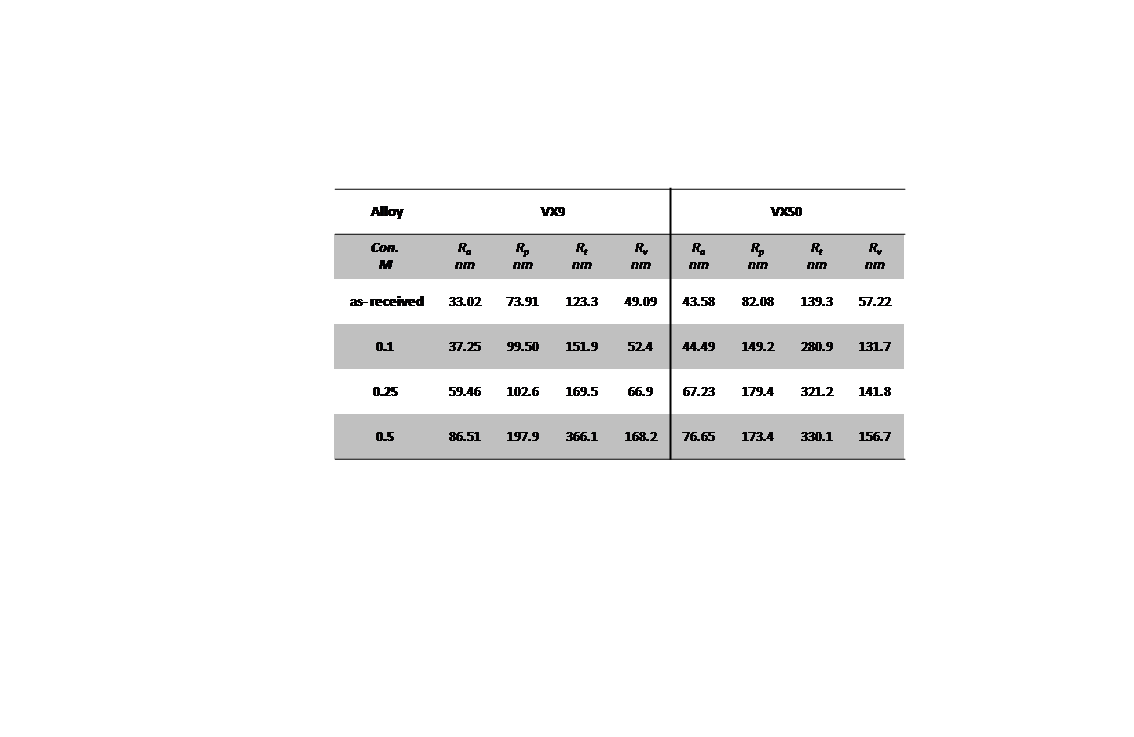

Supplement: S3 Table — (TIF) [file pone.0187567.s003.tif]
